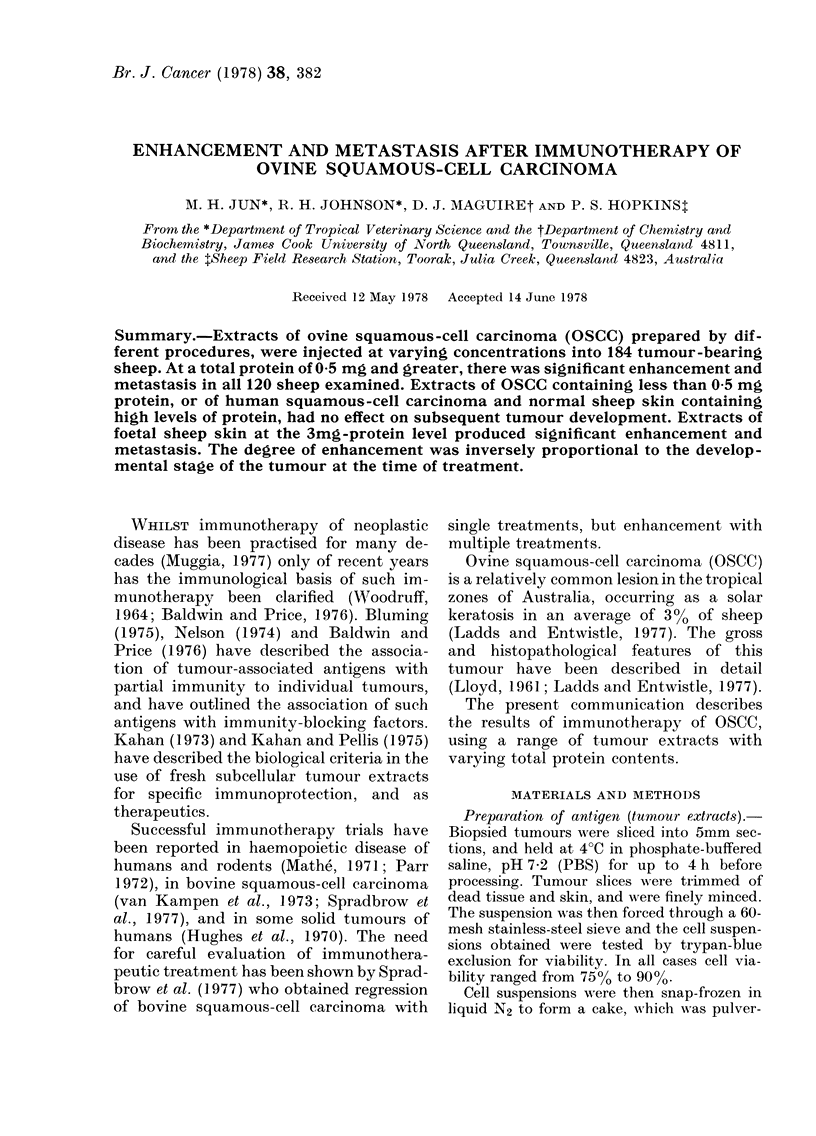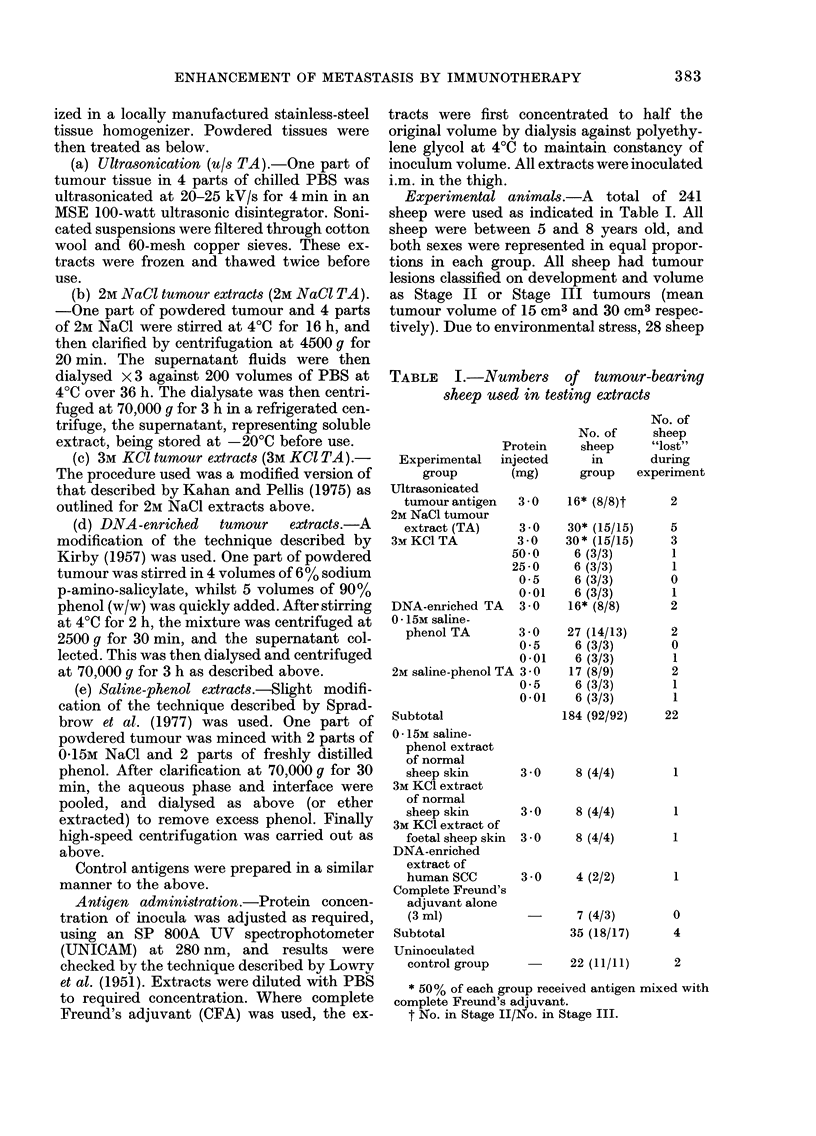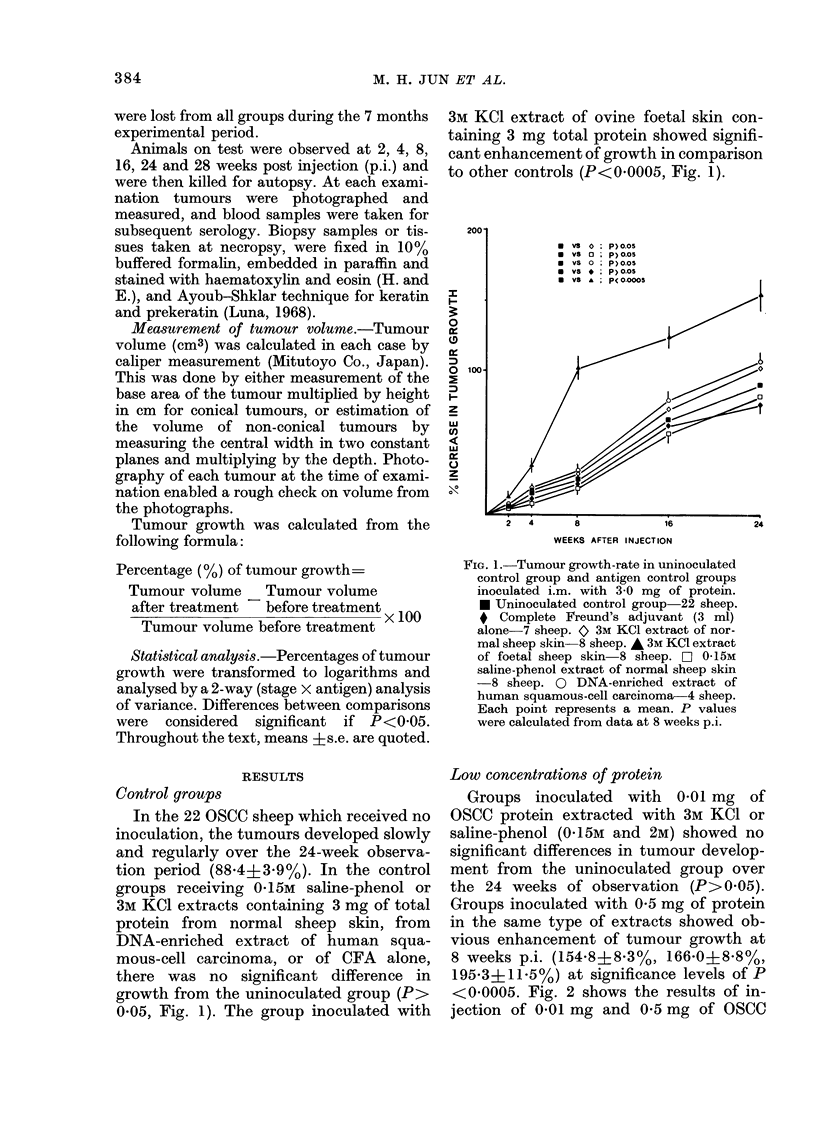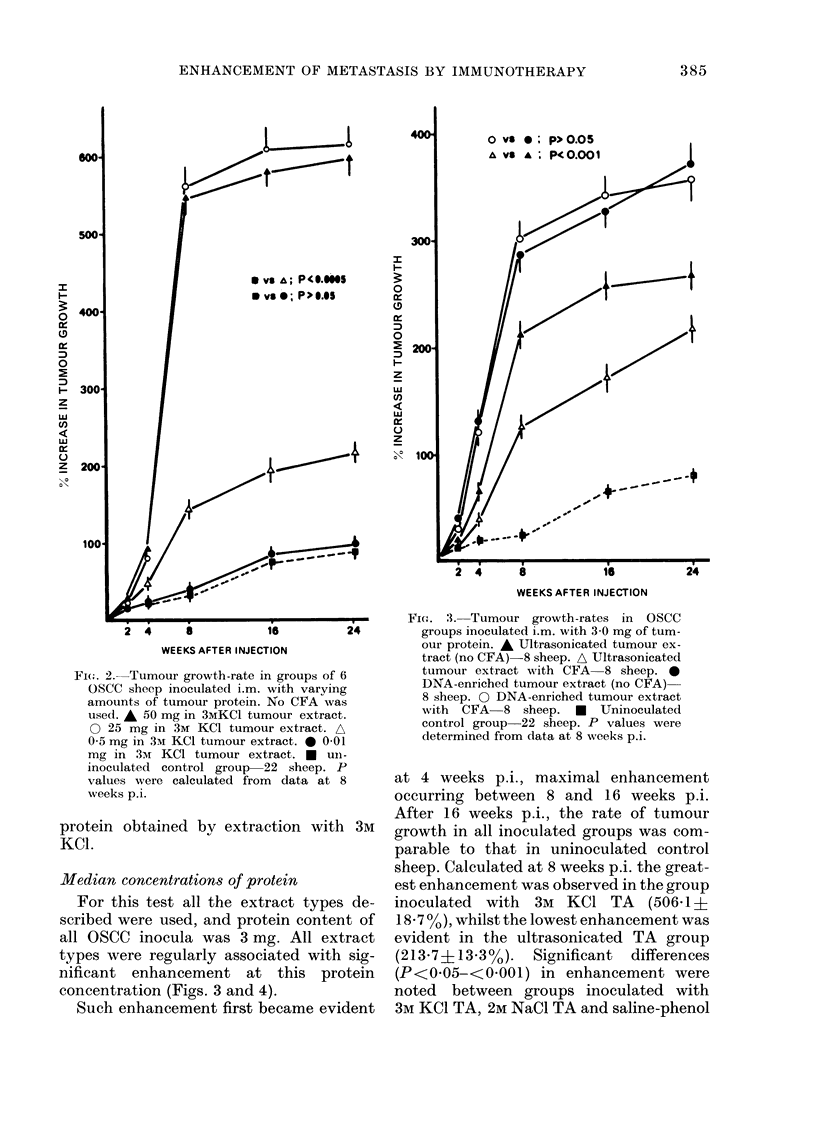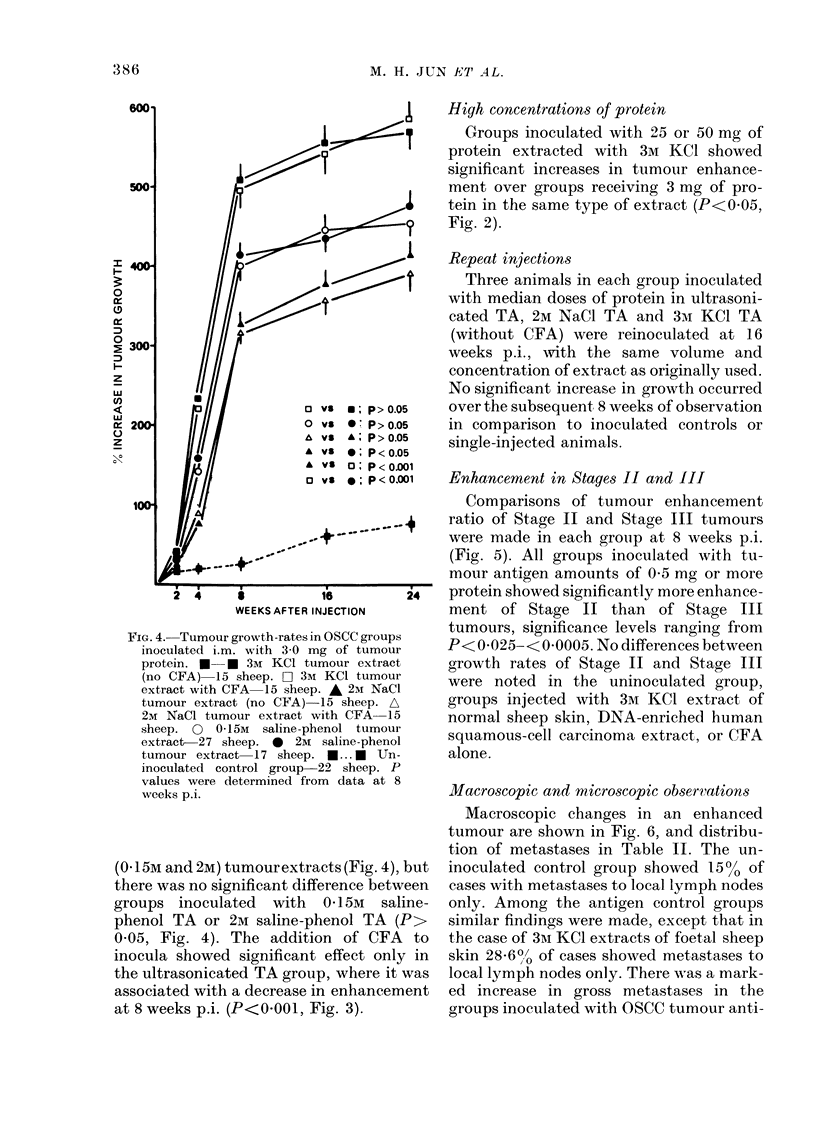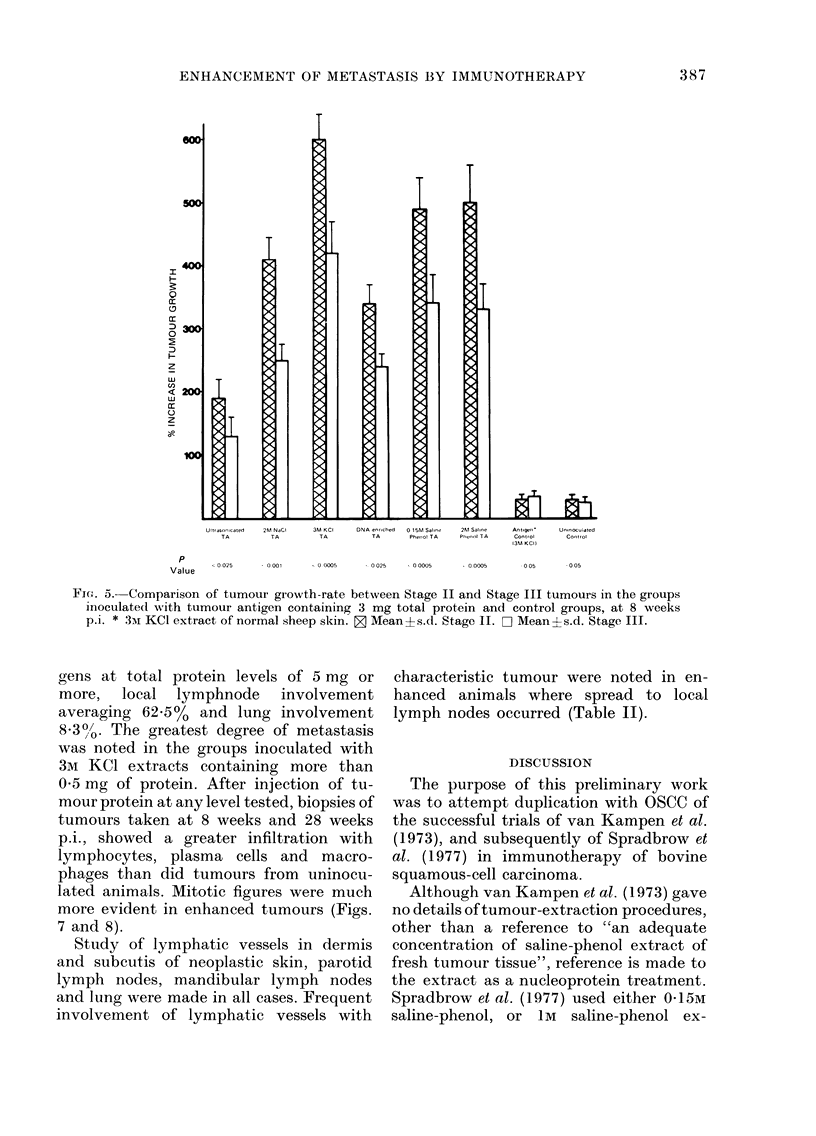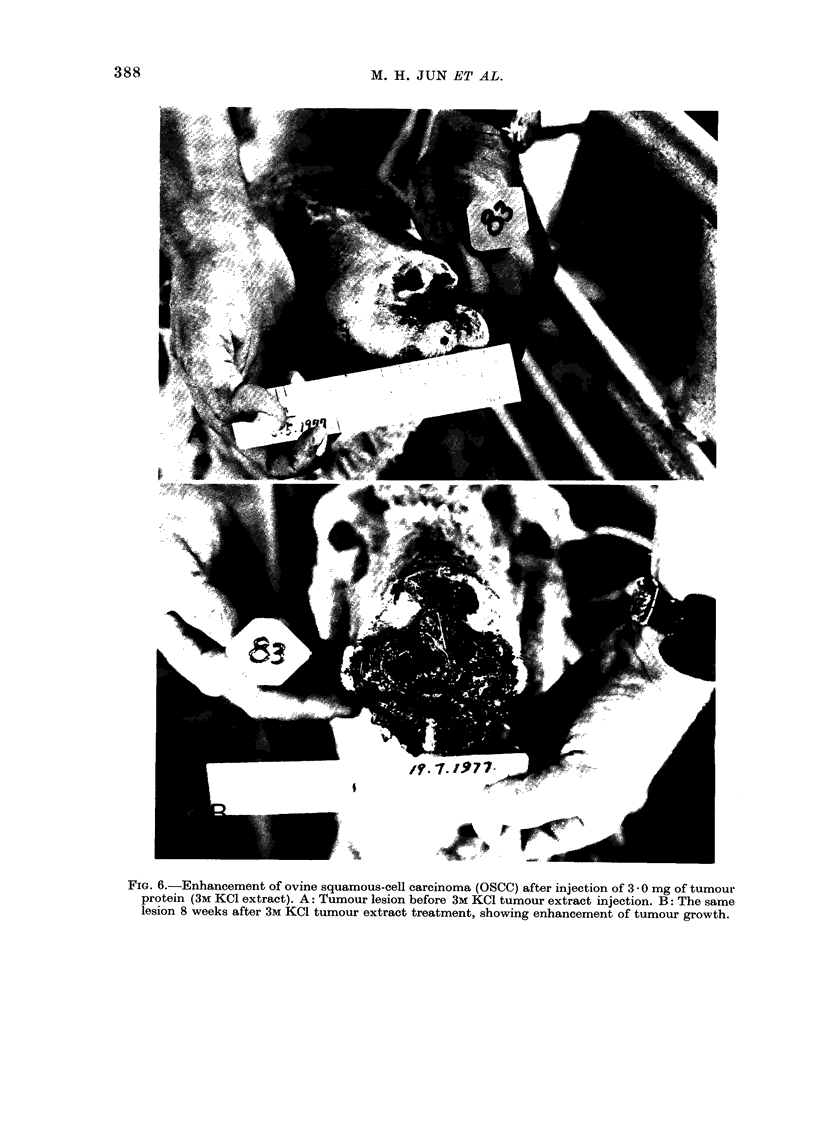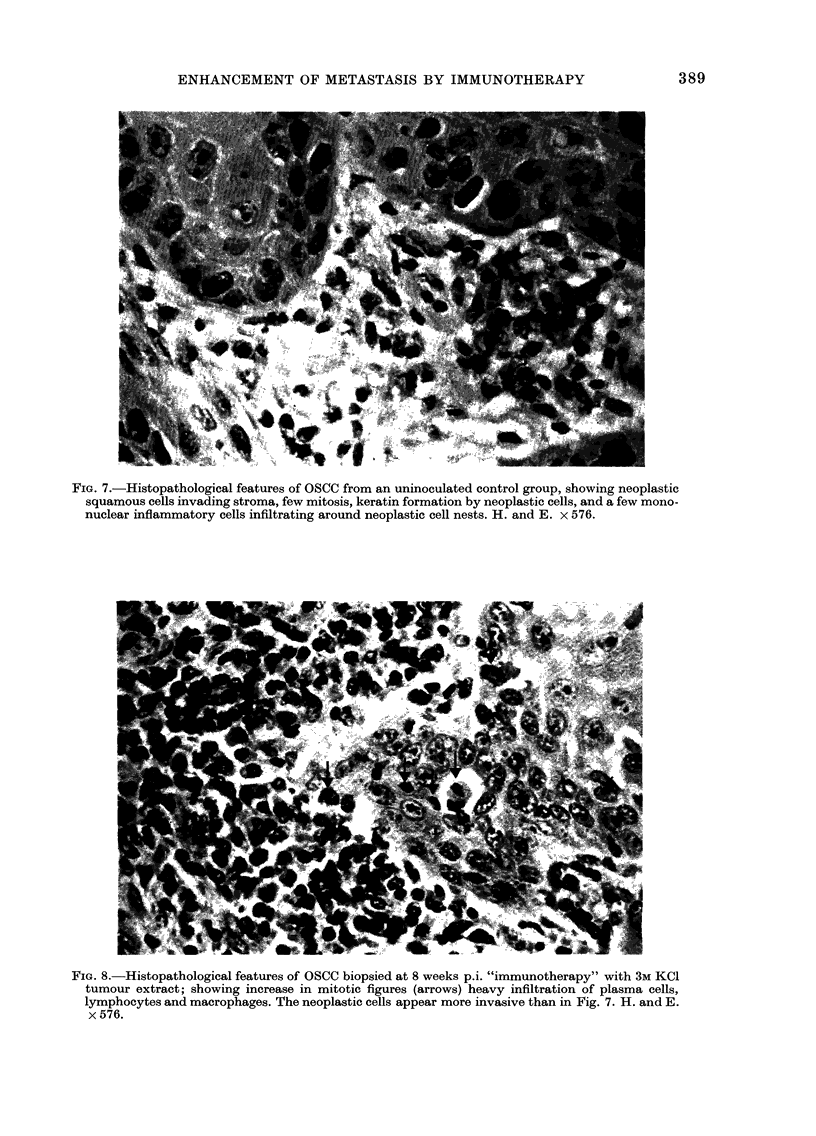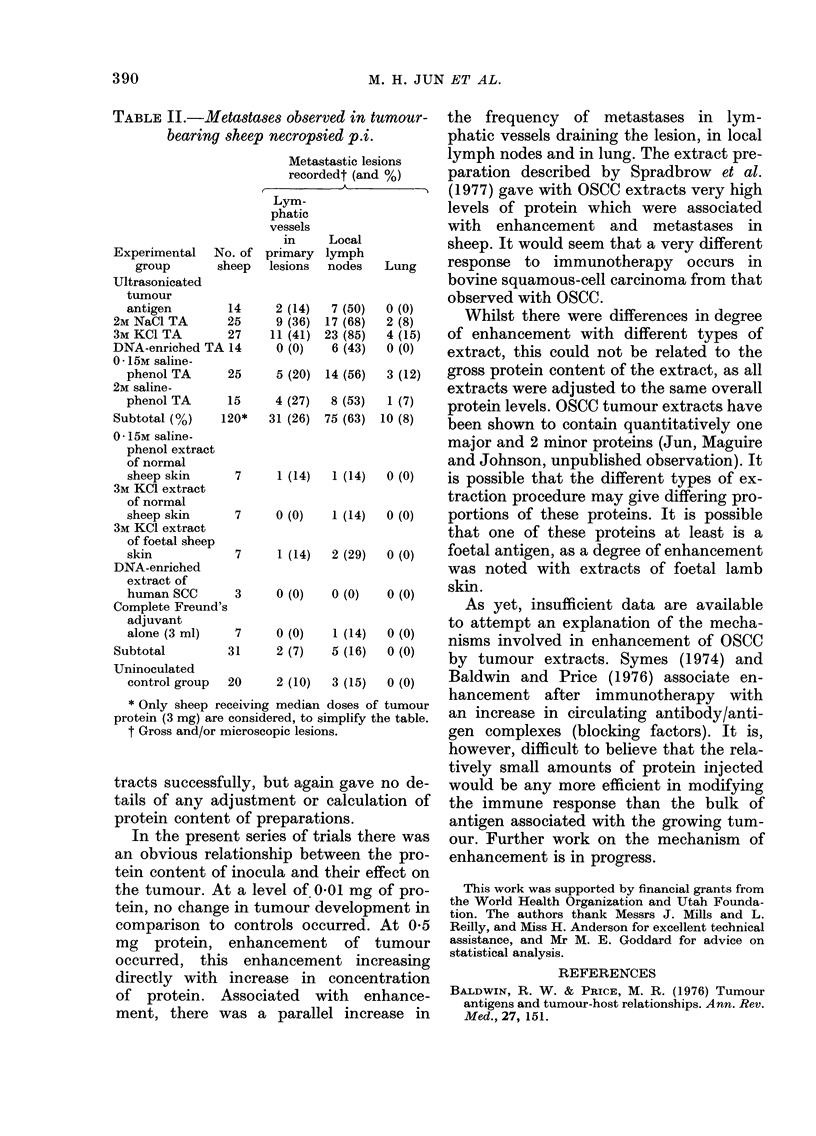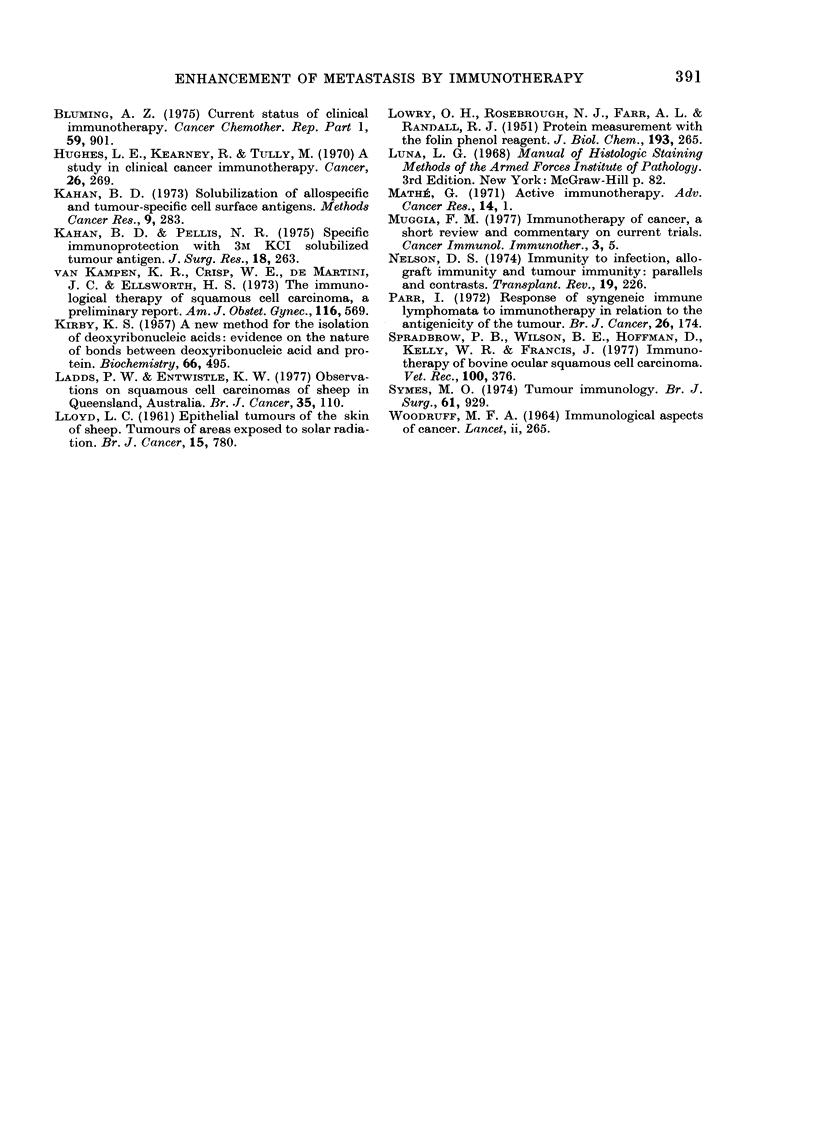# Enhancement and metastasis after immunotherapy of ovine squamous-cell carcinoma.

**DOI:** 10.1038/bjc.1978.218

**Published:** 1978-09

**Authors:** M. H. Jun, R. H. Johnson, D. J. Maguire, P. S. Hopkins

## Abstract

**Images:**


*Br. J. Cancer* (1978) **38**, 382

### ENHANCEMENT AND METASTASIS AFTER IMMUNOTHERAPY OF OVINE SQUAMOUS-CELL CARCINOMA

M. H. JUN*, R. H. JOHNSON*, D. J. MAGUIRE† AND P. S. HOPKINS‡

*From the *Department of Tropical Veterinary Science and the †Department of Chemistry and Biochemistry, James Cook University of North Queensland, Townsville, Queensland* 4811, *and the ‡Sheep Field Research Station, Toorak, Julia Creek, Queensland* 4823, *Australia*

Received 12 May 1978 Accepted 14 June 1978

**Summary.—Extracts of ovine squamous-cell carcinoma (OSCC) prepared by different procedures, were injected at varying concentrations into 184 tumour-bearing sheep. At a total protein of 0·5 mg and greater, there was significant enhancement and metastasis in all 120 sheep examined. Extracts of OSCC containing less than 0·5 mg protein, or of human squamous-cell carcinoma and normal sheep skin containing high levels of protein, had no effect on subsequent tumour development. Extracts of foetal sheep skin at the 3mg-protein level produced significant enhancement and metastasis. The degree of enhancement was inversely proportional to the developmental stage of the tumour at the time of treatment.**

WHILST immunotherapy of neoplastic disease has been practised for many decades (Muggia, 1977) only of recent years has the immunological basis of such immunotherapy been clarified (Woodruff, 1964; Baldwin and Price, 1976). Bluming (1975), Nelson (1974) and Baldwin and Price (1976) have described the association of tumour-associated antigens with partial immunity to individual tumours, and have outlined the association of such antigens with immunity-blocking factors. Kahan (1973) and Kahan and Pellis (1975) have described the biological criteria in the use of fresh subcellular tumour extracts for specific immunoprotection, and as therapeutics.

Successful immunotherapy trials have been reported in haemopoietic disease of humans and rodents (Mathé, 1971; Parr 1972), in bovine squamous-cell carcinoma (van Kampen *et al.*, 1973; Spradbrow *et al.*, 1977), and in some solid tumours of humans (Hughes *et al.*, 1970). The need for careful evaluation of immunotherapeutic treatment has been shown by Spradbrow *et al.* (1977) who obtained regression of bovine squamous-cell carcinoma with single treatments, but enhancement with multiple treatments.

Ovine squamous-cell carcinoma (OSCC) is a relatively common lesion in the tropical zones of Australia, occurring as a solar keratosis in an average of 3% of sheep (Ladds and Entwistle, 1977). The gross and histopathological features of this tumour have been described in detail (Lloyd, 1961; Ladds and Entwistle, 1977).

The present communication describes the results of immunotherapy of OSCC, using a range of tumour extracts with varying total protein contents.

### MATERIALS AND METHODS

*Preparation of antigen (tumour extracts)*.—Biopsied tumours were sliced into 5mm sections, and held at 4°C in phosphate-buffered saline, pH 7·2 (PBS) for up to 4 h before processing. Tumour slices were trimmed of dead tissue and skin, and were finely minced. The suspension was then forced through a 60-mesh stainless-steel sieve and the cell suspensions obtained were tested by trypan-blue exclusion for viability. In all cases cell viability ranged from 75% to 90%.

Cell suspensions were then snap-frozen in liquid $N_2$ to form a cake, which was pulverized in a locally manufactured stainless-steel tissue homogenizer. Powdered tissues were then treated as below.

(a) *Ultrasonication* (*u/s TA*).—One part of tumour tissue in 4 parts of chilled PBS was ultrasonicated at 20–25 kV/s for 4 min in an MSE 100-watt ultrasonic disintegrator. Sonicated suspensions were filtered through cotton wool and 60-mesh copper sieves. These extracts were frozen and thawed twice before use.

(b) 2M *NaCl tumour extracts* (2M *NaCl TA*).—One part of powdered tumour and 4 parts of 2M NaCl were stirred at 4°C for 16 h, and then clarified by centrifugation at 4500 *g* for 20 min. The supernatant fluids were then dialysed ×3 against 200 volumes of PBS at 4°C over 36 h. The dialysate was then centrifuged at 70,000 *g* for 3 h in a refrigerated centrifuge, the supernatant, representing soluble extract, being stored at −20°C before use.

(c) 3M *KCl tumour extracts* (3M *KCl TA*).—The procedure used was a modified version of that described by Kahan and Pellis (1975) as outlined for 2M NaCl extracts above.

(d) *DNA-enriched tumour extracts*.—A modification of the technique described by Kirby (1957) was used. One part of powdered tumour was stirred in 4 volumes of 6% sodium p-amino-salicylate, whilst 5 volumes of 90% phenol (w/w) was quickly added. After stirring at 4°C for 2 h, the mixture was centrifuged at 2500 *g* for 30 min, and the supernatant collected. This was then dialysed and centrifuged at 70,000 *g* for 3 h as described above.

(e) *Saline-phenol extracts*.—Slight modification of the technique described by Spradbrow *et al.* (1977) was used. One part of powdered tumour was minced with 2 parts of 0·15M NaCl and 2 parts of freshly distilled phenol. After clarification at 70,000 *g* for 30 min, the aqueous phase and interface were pooled, and dialysed as above (or ether extracted) to remove excess phenol. Finally high-speed centrifugation was carried out as above.

Control antigens were prepared in a similar manner to the above.

*Antigen administration*.—Protein concentration of inocula was adjusted as required, using an SP 800A UV spectrophotometer (UNICAM) at 280 nm, and results were checked by the technique described by Lowry *et al.* (1951). Extracts were diluted with PBS to required concentration. Where complete Freund's adjuvant (CFA) was used, the extracts were first concentrated to half the original volume by dialysis against polyethylene glycol at 4°C to maintain constancy of inoculum volume. All extracts were inoculated i.m. in the thigh.

*Experimental animals*.—A total of 241 sheep were used as indicated in Table I. All sheep were between 5 and 8 years old, and both sexes were represented in equal proportions in each group. All sheep had tumour lesions classified on development and volume as Stage II or Stage III tumours (mean tumour volume of 15 cm³ and 30 cm³ respectively). Due to environmental stress, 28 sheep

TABLE I.—*Numbers of tumour-bearing sheep used in testing extracts*

| Experimental group | Protein injected (mg) | No. of sheep in group | No. of sheep "lost" during experiment |
|---|---|---|---|
| Ultrasonicated tumour antigen | 3·0 | 16* (8/8)† | 2 |
| 2M NaCl tumour extract (TA) | 3·0 | 30* (15/15) | 5 |
| 3M KCl TA | 3·0 | 30* (15/15) | 3 |
| | 50·0 | 6 (3/3) | 1 |
| | 25·0 | 6 (3/3) | 1 |
| | 0·5 | 6 (3/3) | 0 |
| | 0·01 | 6 (3/3) | 1 |
| DNA-enriched TA | 3·0 | 16* (8/8) | 2 |
| 0·15M saline-phenol TA | 3·0 | 27 (14/13) | 2 |
| | 0·5 | 6 (3/3) | 0 |
| | 0·01 | 6 (3/3) | 1 |
| 2M saline-phenol TA | 3·0 | 17 (8/9) | 2 |
| | 0·5 | 6 (3/3) | 1 |
| | 0·01 | 6 (3/3) | 1 |
| Subtotal | | 184 (92/92) | 22 |
| 0·15M saline-phenol extract of normal sheep skin | 3·0 | 8 (4/4) | 1 |
| 3M KCl extract of normal sheep skin | 3·0 | 8 (4/4) | 1 |
| 3M KCl extract of foetal sheep skin | 3·0 | 8 (4/4) | 1 |
| DNA-enriched extract of human SCC | 3·0 | 4 (2/2) | 1 |
| Complete Freund's adjuvant alone (3 ml) | — | 7 (4/3) | 0 |
| Subtotal | | 35 (18/17) | 4 |
| Uninoculated control group | — | 22 (11/11) | 2 |

* 50% of each group received antigen mixed with complete Freund's adjuvant.
† No. in Stage II/No. in Stage III.

were lost from all groups during the 7 months experimental period.

Animals on test were observed at 2, 4, 8, 16, 24 and 28 weeks post injection (p.i.) and were then killed for autopsy. At each examination tumours were photographed and measured, and blood samples were taken for subsequent serology. Biopsy samples or tissues taken at necropsy, were fixed in 10% buffered formalin, embedded in paraffin and stained with haematoxylin and eosin (H. and E.), and Ayoub–Shklar technique for keratin and prekeratin (Luna, 1968).

*Measurement of tumour volume.*—Tumour volume ($cm^3$) was calculated in each case by caliper measurement (Mitutoyo Co., Japan). This was done by either measurement of the base area of the tumour multiplied by height in cm for conical tumours, or estimation of the volume of non-conical tumours by measuring the central width in two constant planes and multiplying by the depth. Photography of each tumour at the time of examination enabled a rough check on volume from the photographs.

Tumour growth was calculated from the following formula:

$$\text{Percentage (\%) of tumour growth} = \frac{\begin{array}{c}\text{Tumour volume}\\ \text{after treatment}\end{array} - \begin{array}{c}\text{Tumour volume}\\ \text{before treatment}\end{array}}{\text{Tumour volume before treatment}} \times 100$$

*Statistical analysis.*—Percentages of tumour growth were transformed to logarithms and analysed by a 2-way (stage × antigen) analysis of variance. Differences between comparisons were considered significant if $P<0{\cdot}05$. Throughout the text, means ±s.e. are quoted.

### RESULTS

### *Control groups*

In the 22 OSCC sheep which received no inoculation, the tumours developed slowly and regularly over the 24-week observation period (88·4±3·9%). In the control groups receiving 0·15M saline-phenol or 3M KCl extracts containing 3 mg of total protein from normal sheep skin, from DNA-enriched extract of human squamous-cell carcinoma, or of CFA alone, there was no significant difference in growth from the uninoculated group ($P>0{\cdot}05$, Fig. 1). The group inoculated with 3M KCl extract of ovine foetal skin containing 3 mg total protein showed significant enhancement of growth in comparison to other controls ($P<0{\cdot}0005$, Fig. 1).

FIG. 1.—Tumour growth-rate in uninoculated control group and antigen control groups inoculated i.m. with 3·0 mg of protein. ■ Uninoculated control group—22 sheep. ♦ Complete Freund's adjuvant (3 ml) alone—7 sheep. ◇ 3M KCl extract of normal sheep skin—8 sheep. ▲ 3M KCl extract of foetal sheep skin—8 sheep. □ 0·15M saline-phenol extract of normal sheep skin —8 sheep. ○ DNA-enriched extract of human squamous-cell carcinoma—4 sheep. Each point represents a mean. *P* values were calculated from data at 8 weeks p.i.

### *Low concentrations of protein*

Groups inoculated with 0·01 mg of OSCC protein extracted with 3M KCl or saline-phenol (0·15M and 2M) showed no significant differences in tumour development from the uninoculated group over the 24 weeks of observation ($P>0{\cdot}05$). Groups inoculated with 0·5 mg of protein in the same type of extracts showed obvious enhancement of tumour growth at 8 weeks p.i. (154·8±8·3%, 166·0±8·8%, 195·3±11·5%) at significance levels of $P<0{\cdot}0005$. Fig. 2 shows the results of injection of 0·01 mg and 0·5 mg of OSCC

FIG. 2.—Tumour growth-rate in groups of 6 OSCC sheep inoculated i.m. with varying amounts of tumour protein. No CFA was used. ▲ 50 mg in 3MKCl tumour extract. ○ 25 mg in 3M KCl tumour extract. △ 0·5 mg in 3M KCl tumour extract. ● 0·01 mg in 3M KCl tumour extract. ■ uninoculated control group—22 sheep. *P* values were calculated from data at 8 weeks p.i.

FIG. 3.—Tumour growth-rates in OSCC groups inoculated i.m. with 3·0 mg of tumour protein. ▲ Ultrasonicated tumour extract (no CFA)—8 sheep. △ Ultrasonicated tumour extract with CFA—8 sheep. ● DNA-enriched tumour extract (no CFA)—8 sheep. ○ DNA-enriched tumour extract with CFA—8 sheep. ■ Uninoculated control group—22 sheep. *P* values were determined from data at 8 weeks p.i.

protein obtained by extraction with 3M KCl.

*Median concentrations of protein*

For this test all the extract types described were used, and protein content of all OSCC inocula was 3 mg. All extract types were regularly associated with significant enhancement at this protein concentration (Figs. 3 and 4).

Such enhancement first became evident at 4 weeks p.i., maximal enhancement occurring between 8 and 16 weeks p.i. After 16 weeks p.i., the rate of tumour growth in all inoculated groups was comparable to that in uninoculated control sheep. Calculated at 8 weeks p.i. the greatest enhancement was observed in the group inoculated with 3M KCl TA (506·1 ± 18·7%), whilst the lowest enhancement was evident in the ultrasonicated TA group (213·7 ± 13·3%). Significant differences ($P$<0·05–<0·001) in enhancement were noted between groups inoculated with 3M KCl TA, 2M NaCl TA and saline-phenol

FIG. 4.—Tumour growth-rates in OSCC groups inoculated i.m. with 3·0 mg of tumour protein. ■—■ 3M KCl tumour extract (no CFA)—15 sheep. □ 3M KCl tumour extract with CFA—15 sheep. ▲ 2M NaCl tumour extract (no CFA)—15 sheep. △ 2M NaCl tumour extract with CFA—15 sheep. ○ 0·15M saline-phenol tumour extract—27 sheep. ● 2M saline-phenol tumour extract—17 sheep. ■...■ Uninoculated control group—22 sheep. $P$ values were determined from data at 8 weeks p.i.

(0·15M and 2M) tumour extracts (Fig. 4), but there was no significant difference between groups inoculated with 0·15M saline-phenol TA or 2M saline-phenol TA ($P>0{\cdot}05$, Fig. 4). The addition of CFA to inocula showed significant effect only in the ultrasonicated TA group, where it was associated with a decrease in enhancement at 8 weeks p.i. ($P<0{\cdot}001$, Fig. 3).

*High concentrations of protein*

Groups inoculated with 25 or 50 mg of protein extracted with 3M KCl showed significant increases in tumour enhancement over groups receiving 3 mg of protein in the same type of extract ($P<0{\cdot}05$, Fig. 2).

*Repeat injections*

Three animals in each group inoculated with median doses of protein in ultrasonicated TA, 2M NaCl TA and 3M KCl TA (without CFA) were reinoculated at 16 weeks p.i., with the same volume and concentration of extract as originally used. No significant increase in growth occurred over the subsequent 8 weeks of observation in comparison to inoculated controls or single-injected animals.

*Enhancement in Stages II and III*

Comparisons of tumour enhancement ratio of Stage II and Stage III tumours were made in each group at 8 weeks p.i. (Fig. 5). All groups inoculated with tumour antigen amounts of 0·5 mg or more protein showed significantly more enhancement of Stage II than of Stage III tumours, significance levels ranging from $P<0{\cdot}025$–$<0{\cdot}0005$. No differences between growth rates of Stage II and Stage III were noted in the uninoculated group, groups injected with 3M KCl extract of normal sheep skin, DNA-enriched human squamous-cell carcinoma extract, or CFA alone.

*Macroscopic and microscopic observations*

Macroscopic changes in an enhanced tumour are shown in Fig. 6, and distribution of metastases in Table II. The uninoculated control group showed 15% of cases with metastases to local lymph nodes only. Among the antigen control groups similar findings were made, except that in the case of 3M KCl extracts of foetal sheep skin 28·6% of cases showed metastases to local lymph nodes only. There was a marked increase in gross metastases in the groups inoculated with OSCC tumour anti-

FIG. 5.—Comparison of tumour growth-rate between Stage II and Stage III tumours in the groups inoculated with tumour antigen containing 3 mg total protein and control groups, at 8 weeks p.i. * 3M KCl extract of normal sheep skin. ☒ Mean ± s.d. Stage II. ☐ Mean ± s.d. Stage III.

gens at total protein levels of 5 mg or more, local lymphnode involvement averaging 62·5% and lung involvement 8·3%. The greatest degree of metastasis was noted in the groups inoculated with 3M KCl extracts containing more than 0·5 mg of protein. After injection of tumour protein at any level tested, biopsies of tumours taken at 8 weeks and 28 weeks p.i., showed a greater infiltration with lymphocytes, plasma cells and macrophages than did tumours from uninoculated animals. Mitotic figures were much more evident in enhanced tumours (Figs. 7 and 8).

Study of lymphatic vessels in dermis and subcutis of neoplastic skin, parotid lymph nodes, mandibular lymph nodes and lung were made in all cases. Frequent involvement of lymphatic vessels with characteristic tumour were noted in enhanced animals where spread to local lymph nodes occurred (Table II).

### DISCUSSION

The purpose of this preliminary work was to attempt duplication with OSCC of the successful trials of van Kampen *et al.* (1973), and subsequently of Spradbrow *et al.* (1977) in immunotherapy of bovine squamous-cell carcinoma.

Although van Kampen *et al.* (1973) gave no details of tumour-extraction procedures, other than a reference to "an adequate concentration of saline-phenol extract of fresh tumour tissue", reference is made to the extract as a nucleoprotein treatment. Spradbrow *et al.* (1977) used either 0·15M saline-phenol, or 1M saline-phenol ex-

FIG. 6.—Enhancement of ovine squamous-cell carcinoma (OSCC) after injection of 3·0 mg of tumour protein (3M KCl extract). A: Tumour lesion before 3M KCl tumour extract injection. B: The same lesion 8 weeks after 3M KCl tumour extract treatment, showing enhancement of tumour growth.

FIG. 7.—Histopathological features of OSCC from an uninoculated control group, showing neoplastic squamous cells invading stroma, few mitosis, keratin formation by neoplastic cells, and a few mononuclear inflammatory cells infiltrating around neoplastic cell nests. H. and E. ×576.

FIG. 8.—Histopathological features of OSCC biopsied at 8 weeks p.i. "immunotherapy" with 3M KCl tumour extract; showing increase in mitotic figures (arrows) heavy infiltration of plasma cells, lymphocytes and macrophages. The neoplastic cells appear more invasive than in Fig. 7. H. and E. ×576.

TABLE II.—*Metastases observed in tumour-bearing sheep necropsied p.i.*

| Experimental group | No. of sheep | Metastastic lesions recorded† (and %): Lymphatic vessels in primary lesions | Local lymph nodes | Lung |
|---|---|---|---|---|
| Ultrasonicated tumour antigen | 14 | 2 (14) | 7 (50) | 0 (0) |
| 2M NaCl TA | 25 | 9 (36) | 17 (68) | 2 (8) |
| 3M KCl TA | 27 | 11 (41) | 23 (85) | 4 (15) |
| DNA-enriched TA | 14 | 0 (0) | 6 (43) | 0 (0) |
| 0·15M saline-phenol TA | 25 | 5 (20) | 14 (56) | 3 (12) |
| 2M saline-phenol TA | 15 | 4 (27) | 8 (53) | 1 (7) |
| Subtotal (%) | 120* | 31 (26) | 75 (63) | 10 (8) |
| 0·15M saline-phenol extract of normal sheep skin | 7 | 1 (14) | 1 (14) | 0 (0) |
| 3M KCl extract of normal sheep skin | 7 | 0 (0) | 1 (14) | 0 (0) |
| 3M KCl extract of foetal sheep skin | 7 | 1 (14) | 2 (29) | 0 (0) |
| DNA-enriched extract of human SCC | 3 | 0 (0) | 0 (0) | 0 (0) |
| Complete Freund's adjuvant alone (3 ml) | 7 | 0 (0) | 1 (14) | 0 (0) |
| Subtotal | 31 | 2 (7) | 5 (16) | 0 (0) |
| Uninoculated control group | 20 | 2 (10) | 3 (15) | 0 (0) |

* Only sheep receiving median doses of tumour protein (3 mg) are considered, to simplify the table.
† Gross and/or microscopic lesions.

tracts successfully, but again gave no details of any adjustment or calculation of protein content of preparations.

In the present series of trials there was an obvious relationship between the protein content of inocula and their effect on the tumour. At a level of 0·01 mg of protein, no change in tumour development in comparison to controls occurred. At 0·5 mg protein, enhancement of tumour occurred, this enhancement increasing directly with increase in concentration of protein. Associated with enhancement, there was a parallel increase in the frequency of metastases in lymphatic vessels draining the lesion, in local lymph nodes and in lung. The extract preparation described by Spradbrow *et al.* (1977) gave with OSCC extracts very high levels of protein which were associated with enhancement and metastases in sheep. It would seem that a very different response to immunotherapy occurs in bovine squamous-cell carcinoma from that observed with OSCC.

Whilst there were differences in degree of enhancement with different types of extract, this could not be related to the gross protein content of the extract, as all extracts were adjusted to the same overall protein levels. OSCC tumour extracts have been shown to contain quantitatively one major and 2 minor proteins (Jun, Maguire and Johnson, unpublished observation). It is possible that the different types of extraction procedure may give differing proportions of these proteins. It is possible that one of these proteins at least is a foetal antigen, as a degree of enhancement was noted with extracts of foetal lamb skin.

As yet, insufficient data are available to attempt an explanation of the mechanisms involved in enhancement of OSCC by tumour extracts. Symes (1974) and Baldwin and Price (1976) associate enhancement after immunotherapy with an increase in circulating antibody/antigen complexes (blocking factors). It is, however, difficult to believe that the relatively small amounts of protein injected would be any more efficient in modifying the immune response than the bulk of antigen associated with the growing tumour. Further work on the mechanism of enhancement is in progress.

This work was supported by financial grants from the World Health Organization and Utah Foundation. The authors thank Messrs J. Mills and L. Reilly, and Miss H. Anderson for excellent technical assistance, and Mr M. E. Goddard for advice on statistical analysis.

REFERENCES

BALDWIN, R. W. & PRICE, M. R. (1976) Tumour antigens and tumour-host relationships. *Ann. Rev. Med.*, **27**, 151.

BLUMING, A. Z. (1975) Current status of clinical immunotherapy. *Cancer Chemother. Rep. Part* 1, **59**, 901.

HUGHES, L. E., KEARNEY, R. & TULLY, M. (1970) A study in clinical cancer immunotherapy. *Cancer*, **26**, 269.

KAHAN, B. D. (1973) Solubilization of allospecific and tumour-specific cell surface antigens. *Methods Cancer Res.*, **9**, 283.

KAHAN, B. D. & PELLIS, N. R. (1975) Specific immunoprotection with 3M KCI solubilized tumour antigen. *J. Surg. Res.*, **18**, 263.

VAN KAMPEN, K. R., CRISP, W. E., DE MARTINI, J. C. & ELLSWORTH, H. S. (1973) The immunological therapy of squamous cell carcinoma, a preliminary report. *Am. J. Obstet. Gynec.*, **116**, 569.

KIRBY, K. S. (1957) A new method for the isolation of deoxyribonucleic acids: evidence on the nature of bonds between deoxyribonucleic acid and protein. *Biochemistry*, **66**, 495.

LADDS, P. W. & ENTWISTLE, K. W. (1977) Observations on squamous cell carcinomas of sheep in Queensland, Australia. *Br. J. Cancer*, **35**, 110.

LLOYD, L. C. (1961) Epithelial tumours of the skin of sheep. Tumours of areas exposed to solar radiation. *Br. J. Cancer*, **15**, 780.

LOWRY, O. H., ROSEBROUGH, N. J., FARR, A. L. & RANDALL, R. J. (1951) Protein measurement with the folin phenol reagent. *J. Biol. Chem.*, **193**, 265.

LUNA, L. G. (1968) *Manual of Histologic Staining Methods of the Armed Forces Institute of Pathology.* 3rd Edition. New York: McGraw-Hill p. 82.

MATHÉ, G. (1971) Active immunotherapy. *Adv. Cancer Res.*, **14**, 1.

MUGGIA, F. M. (1977) Immunotherapy of cancer, a short review and commentary on current trials. *Cancer Immunol. Immunother.*, **3**, 5.

NELSON, D. S. (1974) Immunity to infection, allograft immunity and tumour immunity: parallels and contrasts. *Transplant. Rev.*, **19**, 226.

PARR, I. (1972) Response of syngeneic immune lymphomata to immunotherapy in relation to the antigenicity of the tumour. *Br. J. Cancer*, **26**, 174.

SPRADBROW, P. B., WILSON, B. E., HOFFMAN, D., KELLY, W. R. & FRANCIS, J. (1977) Immunotherapy of bovine ocular squamous cell carcinoma. *Vet. Rec.*, **100**, 376.

SYMES, M. O. (1974) Tumour immunology. *Br. J. Surg.*, **61**, 929.

WOODRUFF, M. F. A. (1964) Immunological aspects of cancer. *Lancet*, ii, 265.